# Supplementary material for: Crop growth dynamics: Fast automatic analysis of LiDAR images in field-plot experiments by specialized software ALFA
Source: PLoS One. 2024 Jan 18;19(1):e0297153. doi: 10.1371/journal.pone.0297153 (PMC10796001; doi:10.1371/journal.pone.0297153)
Supplement: Attachment — Submitted filename: PONE-D-23-28678_reviewer.pdf [file pone.0297153.s001.pdf]

PLOS ONE

ALFA - Airborne LiDAR Field-plot Analysis. Fast automatic point-cloud filtering algorithm for analysis of crop growth dynamics in field-plot experiments.

--Manuscript Draft--

**Manuscript Number:**	PONE-D-23-28678
**Article Type:**	Research Article
**Full Title:**	ALFA - Airborne LiDAR Field-plot Analysis. Fast automatic point-cloud filtering algorithm for analysis of crop growth dynamics in field-plot experiments.
**Short Title:**	ALFA - Airborne LiDAR Field-plot Analysis Algorithm
**Corresponding Author:**	Jan F. Humplik<br>Palacký University in Olomouc<br>Olomouc, CZECH REPUBLIC
**Keywords:**	crop height; plant growth dynamics; UAV LIDAR; point-cloud; Python; field-plot
**Abstract:**	Repeated measurements of crop height to observe plant growth dynamics in real field conditions represent a challenging task. Although there are ways to collect data using sensors on UAV systems, proper data processing and analysis are the key to reliable results. Here we present a fast algorithm ALFA for the processing of UAV LiDAR derived point-clouds to extract the information on crop height at many individual cereal field-plots at multiple time points. Seven scanning flights were performed over 3 blocks of experimental barley field plots between April and June 2021. Resulting point-clouds were processed by the new algorithm ALFA. The software converts point-cloud data into a digital image and extracts the traits of interest – the median crop height at individual field plots. The processing time was about 2 to 3 minutes on a standard PC for a single point-cloud of ca. 100 million points. Three different ways of crop-height data visualization are provided by the software to enable further analysis of the variability in growth parameters. ALFA software is a fast and reliable tool for extraction of plant height in individual field-plots that are often used in agricultural research or plant breeding. We offer this tool freely to the scientific community for non-commercial use.
**Order of Authors:**	Tadeáš Fryčák
	Tomáš Fürst, Dr.
	Radoslav Koprna, Dr.
	Zdeněk Špíšek, Dr.
	Jakub Miřijovsky, Dr.
	Jan F. Humplík, Dr.
**Additional Information:**	
**Question**	**Response**
**Financial Disclosure**<br><br>Enter a financial disclosure statement that describes the sources of funding for the work included in this submission. Review the submission guidelines for detailed requirements. View published research articles from *PLOS ONE* for specific examples.<br><br>This statement is required for submission and **will appear in the published article** if	The work was supported by the ERDF project "Plants as a tool for sustainable global development" (No. CZ.02.1.01/0.0/0.0/16_019/0000827).

Powered by Editorial Manager® and ProduXion Manager® from Aries Systems Corporation

the submission is accepted. Please make sure it is accurate.<br><br>**Unfunded studies**<br>Enter: *The author(s) received no specific funding for this work.*<br><br>**Funded studies**<br>Enter a statement with the following details:<br>• Initials of the authors who received each award<br>• Grant numbers awarded to each author<br>• The full name of each funder<br>• URL of each funder website<br>• Did the sponsors or funders play any role in the study design, data collection and analysis, decision to publish, or preparation of the manuscript?<br>• **NO** - Include this sentence at the end of your statement: *The funders had no role in study design, data collection and analysis, decision to publish, or preparation of the manuscript.*<br>• **YES** - Specify the role(s) played.<br><br>* typeset	
**Competing Interests**<br><br>Use the instructions below to enter a competing interest statement for this submission. On behalf of all authors, disclose any competing interests that could be perceived to bias this work—acknowledging all financial support and any other relevant financial or non-financial competing interests.<br><br>This statement is required for submission and **will appear in the published article** if the submission is accepted. Please make sure it is accurate and that any funding sources listed in your Funding Information later in the submission form are also declared in your Financial Disclosure statement.<br><br>View published research articles from *PLOS ONE* for specific examples.	The authors have declared that no competing interests exist.

Powered by Editorial Manager® and ProduXion Manager® from Aries Systems Corporation

**NO authors have competing interests**<br><br>Enter: *The authors have declared that no competing interests exist.*<br><br>**Authors with competing interests**<br><br>Enter competing interest details beginning with this statement:<br><br>*I have read the journal's policy and the authors of this manuscript have the following competing interests: [insert competing interests here]*<br><br>* typeset	
**Ethics Statement**<br><br>Enter an ethics statement for this submission. This statement is required if the study involved:<br><br>• Human participants<br>• Human specimens or tissue<br>• Vertebrate animals or cephalopods<br>• Vertebrate embryos or tissues<br>• Field research<br><br>Write "N/A" if the submission does not require an ethics statement.<br><br>General guidance is provided below. Consult the submission guidelines for detailed instructions. **Make sure that all information entered here is included in the Methods section of the manuscript.**	N/A

**Format for specific study types**<br><br>**Human Subject Research (involving human participants and/or tissue)**<br>• Give the name of the institutional review board or ethics committee that approved the study<br>• Include the approval number and/or a statement indicating approval of this research<br>• Indicate the form of consent obtained (written/oral) or the reason that consent was not obtained (e.g. the data were analyzed anonymously)<br><br>**Animal Research (involving vertebrate animals, embryos or tissues)**<br>• Provide the name of the Institutional Animal Care and Use Committee (IACUC) or other relevant ethics board that reviewed the study protocol, and indicate whether they approved this research or granted a formal waiver of ethical approval<br>• Include an approval number if one was obtained<br>• If the study involved *non-human primates*, add *additional details* about animal welfare and steps taken to ameliorate suffering<br>• If anesthesia, euthanasia, or any kind of animal sacrifice is part of the study, include briefly which substances and/or methods were applied<br><br>**Field Research**<br><br>Include the following details if this study involves the collection of plant, animal, or other materials from a natural setting:<br>• Field permit number<br>• Name of the institution or relevant body that granted permission	
**Data Availability**<br><br>Authors are required to make all data underlying the findings described fully available, without restriction, and from the time of publication. PLOS allows rare exceptions to address legal and ethical concerns. See the PLOS Data Policy and FAQ for detailed information.	Yes - all data are fully available without restriction

A Data Availability Statement describing where the data can be found is required at submission. Your answers to this question constitute the Data Availability Statement and **will be published in the article**, if accepted.<br><br>**Important:** Stating 'data available on request from the author' is not sufficient. If your data are only available upon request, select 'No' for the first question and explain your exceptional situation in the text box.<br><br>Do the authors confirm that all data underlying the findings described in their manuscript are fully available without restriction?	
**Describe where the data may be found in full sentences. If you are copying our sample text, replace any instances of XXX with the appropriate details.**<br><br>• If the data are **held or will be held in a public repository**, include URLs, accession numbers or DOIs. If this information will only be available after acceptance, indicate this by ticking the box below. For example: *All XXX files are available from the XXX database (accession number(s) XXX, XXX.).*<br>• If the data are all contained **within the manuscript and/or Supporting Information files**, enter the following: *All relevant data are within the manuscript and its Supporting Information files.*<br>• If neither of these applies but you are able to provide **details of access elsewhere**, with or without limitations, please do so. For example:<br><br>*Data cannot be shared publicly because of [XXX]. Data are available from the XXX Institutional Data Access / Ethics Committee (contact via XXX) for researchers who meet the criteria for access to confidential data.*<br><br>*The data underlying the results presented in the study are available from (include the name of the third party*	Software tool is freely accessible here: https://github.com/frycaktadeas/alfa<br>The datasets analyzed during the current study are available from the corresponding author on reasonable request.

and contact information or URL).<br>• This text is appropriate if the data are owned by a third party and authors do not have permission to share the data.<br><br>* typeset	
Additional data availability information:	

Manuscript
Click here to access/download;Manuscript;MS_Frycak_2023.docx

TITLE

ALFA - Airborne LiDAR Field-plot Analysis. Fast automatic point-cloud filtering algorithm for analysis of crop growth dynamics in field-plot experiments.

AUTHORS

Fryčák Tadeáš[2], Fürst Tomáš[1], Koprna Radoslav[2], Špíšek Zdeněk[2], Miřijovský Jakub[3], Humplík Jan F.[2*].

AFILIATIONS

1 Department of Mathematical Analysis and Applications of Mathematics, Faculty of Science, Palacký University, 17. Listopadu 50, 771 46 Olomouc, Czech Republic.

2 Department of Chemical Biology, Faculty of Science, Palacký University, Šlechtitelů 27, 78371 Olomouc, Czech Republic.

3 Department of Geoinformatics, Faculty of Science, Palacký University, 17. Listopadu 50, 771 46 Olomouc, Czech Republic.

*corresponding author (jan.humplik@upol.cz)

KEYWORDS

crop height; plant growth dynamics; UAV LIDAR; point-cloud; Python; field-plot

ABSTRACT

Repeated measurements of crop height to observe plant growth dynamics in real field conditions represent a challenging task. Although there are ways to collect data using sensors on UAV systems, proper data processing and analysis are the key to reliable results. Here we present a fast algorithm ALFA for the processing of UAV LiDAR derived point-clouds to extract the information on crop height at many individual cereal field-plots at multiple time points. Seven scanning flights were performed over 3 blocks of experimental barley field plots between April and June 2021. Resulting point-clouds were processed by the new algorithm ALFA. The software converts point-cloud data into a digital image and extracts the traits of interest – the median crop height at individual field plots. The processing time was about 2 to 3 minutes on a standard PC for a single point-cloud of ca. 100 million points. Three different ways of crop-height data visualization are provided by the software to enable further analysis

of the variability in growth parameters. ALFA software is a fast and reliable tool for extraction of plant height in individual field-plots that are often used in agricultural research or plant breeding. We offer this tool freely to the scientific community for non-commercial use.

INTRODUCTION

Intensity of plant growth provides key information about the plant's ability to withstand all possible life situations in various environments. Analysis of plant growth is especially useful when performing agricultural experiments and plant breeding programs. However, manual measurements are very laborious, and widely affected by inter-rater variability. Small field-plots that consist of a single treatment (genotype/variety) are often used as the basic unit in plant and agricultural research. To analyze growth (expressed as the change in the crop height in time), the crop height in individual field-plots is manually assessed by some "ruler" resulting in each unit (field-plot) being represented by a single number (or a few numbers). These numbers are taken to represent the crop height over the entire field-plot, regardless of possible spatial heterogeneity which is often observed in agricultural experiments. To obtain more reliable growth data, analysis of crop height in the entire plot area can be performed (Holman et al., 2016). This assures that variability in crop height formed by thousands of individual plants will be properly reflected in the results. Moreover, when plant growth is measured, several measurements of crop height are needed to cover most of the vegetative period. This advocates for automatic (e.g. UAV-aided) rather than manual approaches.

For these reasons, several remote-sensing approaches have been developed, such as structure-from-motion (SFM) or LiDAR-based solution. SFM techniques are cheaper but highly demanding from the data processing point of view. On the contrary, LiDAR systems are precise and can be analyzed relatively fast however, their prize has limited their implementation so far (Iglhaut et al., 2019). LiDAR solutions are not limited to airborne systems only, they also include terrestrial LiDARs (Brede et al., 2017), LiDARs on various moveable platforms such as tractors or sprayers (French et al., 2016). Both SFM and LiDAR solutions are suitable for generation of crop height models (CHM) derived from point-

cloud files. The term CHM was invented for digital surface models (DSM) specifically applied to crops. CHM is defined as a normalized DSM (nDSM) which results from the subtraction of a digital elevation model (DEM = “ground” points only) from DSM containing other features, in our case plant canopy (Holman et al., 2016; Granshaw 2016).

It has been reported that crop-specific CHM perform better than generalized crop models (ten Harkel et al., 2019). Studies were focused on segmentation of individual plants as is typical for maize (Gao et al., 2022; Wang et al., 2023) or on segmentation of entire field-trial plots in case of other cereals, sorghum or cotton (Hütt et al., 2022; Montzka et al., 2023; Malambo et al., 2018; Liu et al., 2020). The conversion of point-clouds to CHM can be done manually using software for digital terrain model processing (Malambo et al., 2018), but specialized algorithms have also been reported (Zhu et al., 2021). Although ~~the~~ CHM can be efficiently utilized for segmentation of trial field-plots, they have been found insufficient for individual plant segmentation (Koch et al., 2006). For these specific purposes other methods based on direct-point segmentation were developed (Li et al., 2012). Other approaches, such as regional growth (Jin et al., 2018) or voxel-space projection (Wang et al., 2008), utilize features of individual points from the point-cloud as well the spatial relations between the points. Gao et al. (2022) recently proposed a method combining RGB orthophoto to identify seed points in young seedlings of maize for their later segmentation from LiDAR point-cloud using fuzzy based C-means clustering analysis. Our previous algorithm (Polák et al., 2021) was based on direct-point analysis strategy to segment trial field-plots of winter wheat. However, direct-point analysis is highly demanding on computing time. This reduced the applicability of our previous algorithm in datasets containing multiple point-clouds. Thus, we propose a new software based on the CHM strategy. The proposed solution reduces the computation time for a 1GB sized point-cloud file from several hours to several minutes without compromising precision and accuracy. In contrast to the previous algorithm, it can be run efficiently on a standard PC or laptop. The aim of this contribution is to describe the algorithm and show its utility in real conditions.

IMPLEMENTATION

The drone data is presented as a 3D point-cloud format, which consists of a list of [x, y, z] coordinates representing the positions of the recorded points. First, we convert this format into a 2.5D digital image format, allowing for the utilization of fast digital image processing methods. However, this conversion results in a loss of information because in a digital image, a single z-value is associated with each [x, y] coordinate. Nevertheless, for crop height measurement purposes, this transformation is suitable. Additionally, no measured z-values are deleted; instead, all z-values corresponding to the same [x, y] coordinate are averaged as described below.

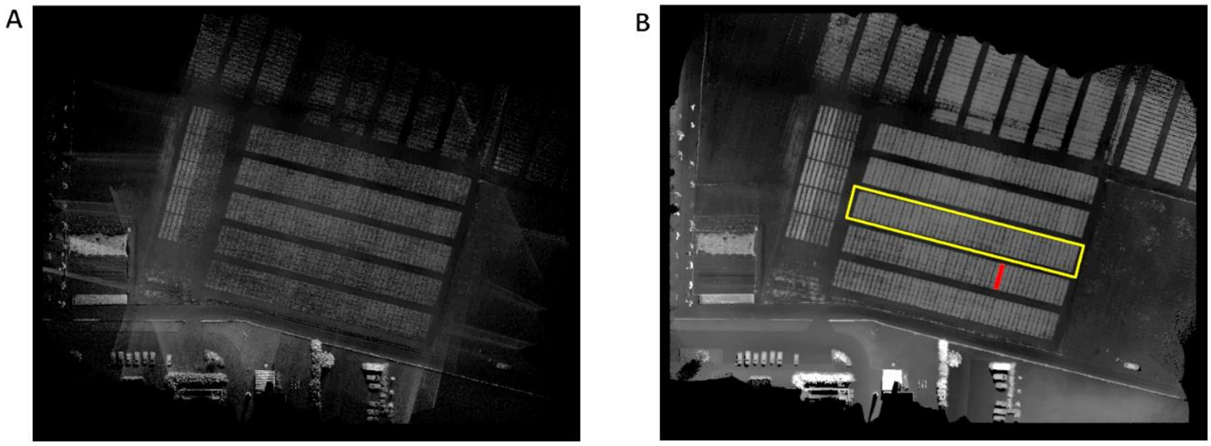

Figure 1 Initial processing of the point-cloud data into the crop height model. A) The raw digital image obtained from the point-cloud data by averaging the z-values corresponding to the same [x, y] coordinate. B) The digital image after the application of the median filter to impute the missing values. A single *block* highlighted in yellow, a single *variant* within the block highlighted in red.

Data filtering and rasterization – The first step involves cleaning the point-cloud data by eliminating any outlier points that fall outside the 99.9% quantile in the x, y, or z coordinates. Subsequently, a uniform rectangular grid is created in the x-y plane, and each 3D point in the point cloud is assigned to the nearest grid pixel. The elevation of each grid pixel is then calculated as the average of all z-coordinates of cloud points assigned to that specific grid point. During this process, the elevation of certain grid points may remain undefined if no cloud points were assigned to them. To address this, a median filter is applied to impute the missing values. Furthermore, the z-coordinates are quantized to optimize the resulting image for a 16-bit depth. After completing these steps, the original 3D point-

cloud files are transformed and saved as significantly smaller 16-bit PNG images, while preserving the crop height information. Each PNG file corresponds to a specific drone measurement time. These PNG images can be conveniently processed using standard image processing tools.

Selection of Region of Interest (ROI) – The initial image from the repeated measurements undergoes rotation and cropping to a ROI, which is easily accomplished by the user through a Graphical User Interface (GUI). Next, all subsequent sequential images are automatically cropped to match the selected ROI, following the same process as the first image in the series.

Instance segmentation algorithm – Subsequently, the ROI undergoes segmentation to identify the individual field plots within the image for later CHM generation. Vertical and horizontal edges separating the field plots are automatically detected by summing the image values vertically and horizontally (Fig 2A). Peaks are automatically detected in the summed images by means of numerical derivatives (Fig 2B).

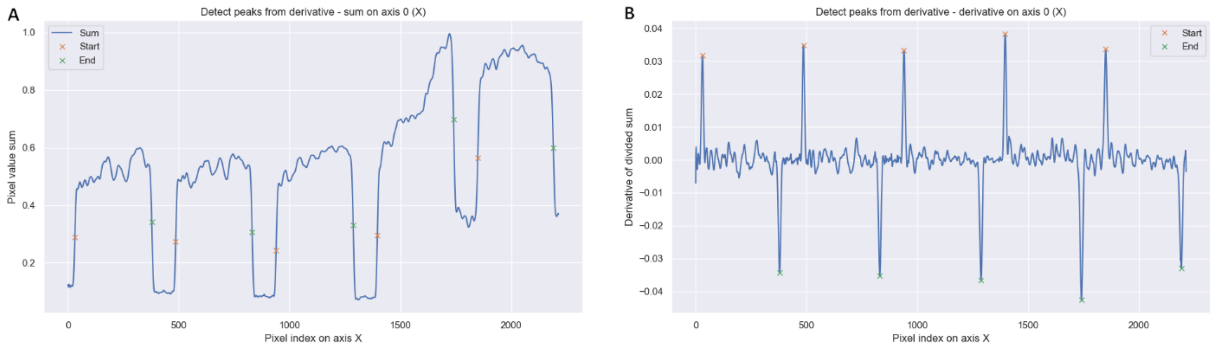

Figure 2 Instance segmentation of individual fields. The digital image of a field block highlighted in yellow in Figure 1B is summed along the shorter edge. A) Vertical normalized sum of the processed image (blue) with automatically identified left (green) and right (orange) edges of the individual field plots. B) First derivative of the sum in panel A, enabling the automatic detection of the plot edges.

Distortion correction – The individual field plots are often thin and long and their images may be skewed. Thus, we apply a correction by fitting a parallelogram to the boundary of the field plots (Fig

3). The deformation is then automatically rectified through an affine transform algorithm, which is accessible within the OpenCV toolbox (OpenCV, 2008).

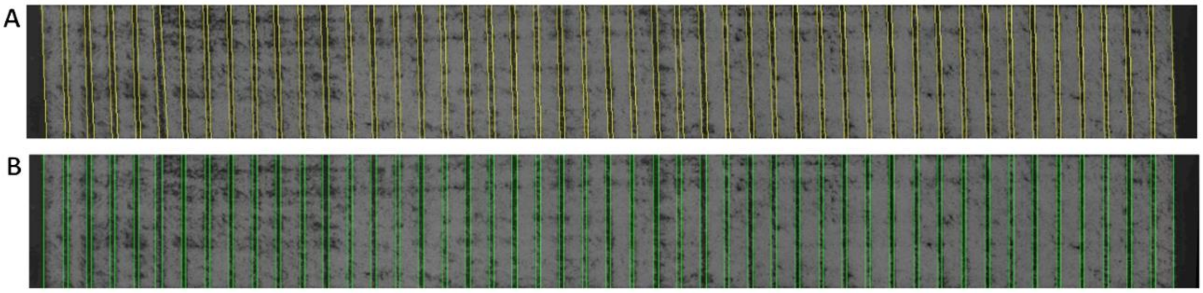

Figure 3 Distortion correction A) A detail of a single *block* before the affine correction. B) The same *block* after the affine correction.

Crop height computation – The digital image is very “noisy” in the sense that z-values corresponding to neighboring grid points may be very different. To create a CHM, a maximum filter is used. Each pixel value in the image is substituted by the maximum of its neighbors. Intuitively, this operation corresponds to covering the original image by a deformable blanket. Next, the ground-surface (DEM) at time zero is subtracted from each image to account for any variability in the terrain. For each field-plot and each time of measurement, the following characteristics of the crop height are computed: mean, standard deviation, median, minimum, maximum, and several quantiles. The relative growth rate between time t_1 and t_2 is computed using the relation (Hoffmann and Poorter, n.d.)

$$RGR(t_1, t_2) = \frac{\ln S(t_2) - \ln S(t_1)}{t_2 - t_1}$$

Where S(t) is the median crop height at time t.

Each field plot is indexed by its *variant* number *i* and *block* number *j* (see Fig 1). The growth in each field-segment is analyzed as follows. Let $S_{ij}(t)$ be the median crop height of variant *i* in block *j* at time *t*. A logistic growth curve is fitted to the data (separately in each field plot). The logistic curve is defined by:

$$S(t) = \frac{A}{1 + exp(-Bt - C)}$$

Where *A* characterizes the maximum height that the crop would reach if observed long enough, *B* captures the growth velocity, and *C* captures growth onset (for review see Tjørve, 2003). The parameters *A, B, C* are found using a standard non-linear least squares regression (implemented in MATLAB). The variability in the parameters among the field-plots can be visualized by means of a heat map (see Fig 4). The effect of various treatments of the crop can be evaluated by analyzing the variability among the A, B and C parameters inferred from the crop heights.

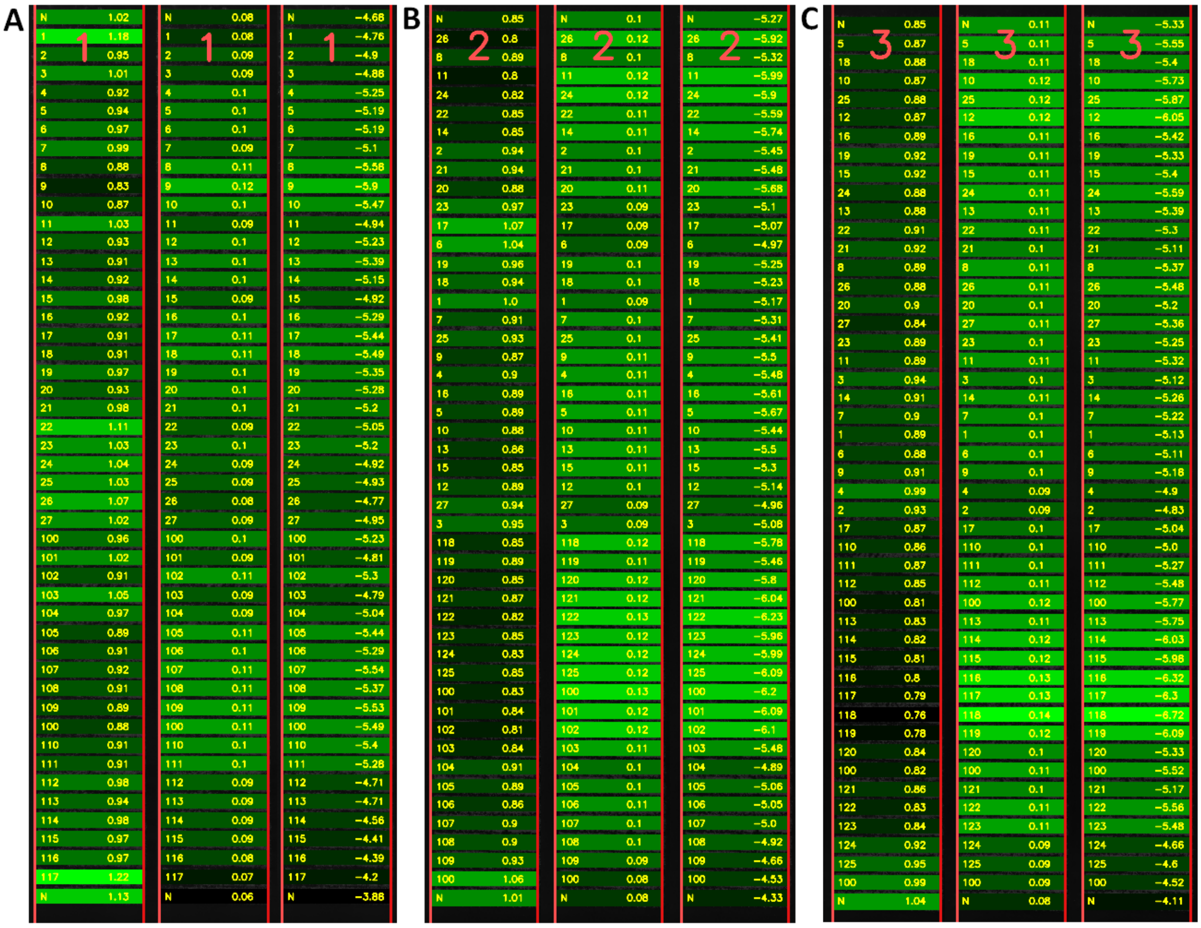

Figure 4 Visualization of the parameters of the sigmoid growth model for individual field-plots. Parameter A (left panel) corresponds to the maximum possible height of the crop. Parameter B (middle panel) captures the "growth velocity" because it corresponds to the slope of the sigmoid curve. Parameter C (right panel) is the "offset" which captures the left-right shift of the sigmoid. Parameters are shown for blocks of field-plots 1, 2 and 3 as indicated by the red numbers in the top row. The left column of numbers stands for the ID of the variant, the right column shows the numerical value of the respective parameter of the sigmoid model. The parameter values are color coded. Observe the spatially correlated variability in the parameters.

RESULTS AND DISCUSSION

Data for software testing and optimization were acquired using UAV LiDAR system (Ricopter VUX-SYS, Riegl GmBh, Austria) using same parameters as described previously (Polák et al., 2021). Eight flights from March to June were performed to scan an array of barley field-plots organized into three experimental blocks (48 variants per each block). The flight altitude was set to 20 m AGL, horizontal speed of flight 4 m/s, scanning line distance 9 m, calculated side overlap (77%, 31 m). The LiDAR sensor was running at the maximum laser pulse rate (550 kHz). LiDAR scans were processed as described previously to point-cloud files in the "las" format. These files were processed by our software to segment point-cloud derived crop height model (CHM) and analyze the crop height at individual field-plots.

Manual validation in selected field-plots

To validate the software and assess the UAV LiDAR scanning accuracy, we performed manual measurement using geodetic GPS and a ruler to measure the crop height. These measurements were performed the same day as the UAV LiDAR scanning and covered 6 and 7 field-plots homogeneously distributed across blocks 1 and 2. We performed validation of the crop height measurement in two blocks 1 and 2 by manually measuring the crop height at 5 points within each field-plot (see Figure 5). Our validation showed that the Root Mean Square Deviation (RMSD) of the software-computed crop height from the manual measurement was 5.7 cm (0.057 m) for both blocks together. The error distribution for each individual block is depicted in Figure 5. This is better than the results reached with the previous software that was validated in wheat field-plots (Polák et al., 2021). The manual measurements are based on the median of 5 values, whereas the LiDAR data reflect the height of the entire plot.

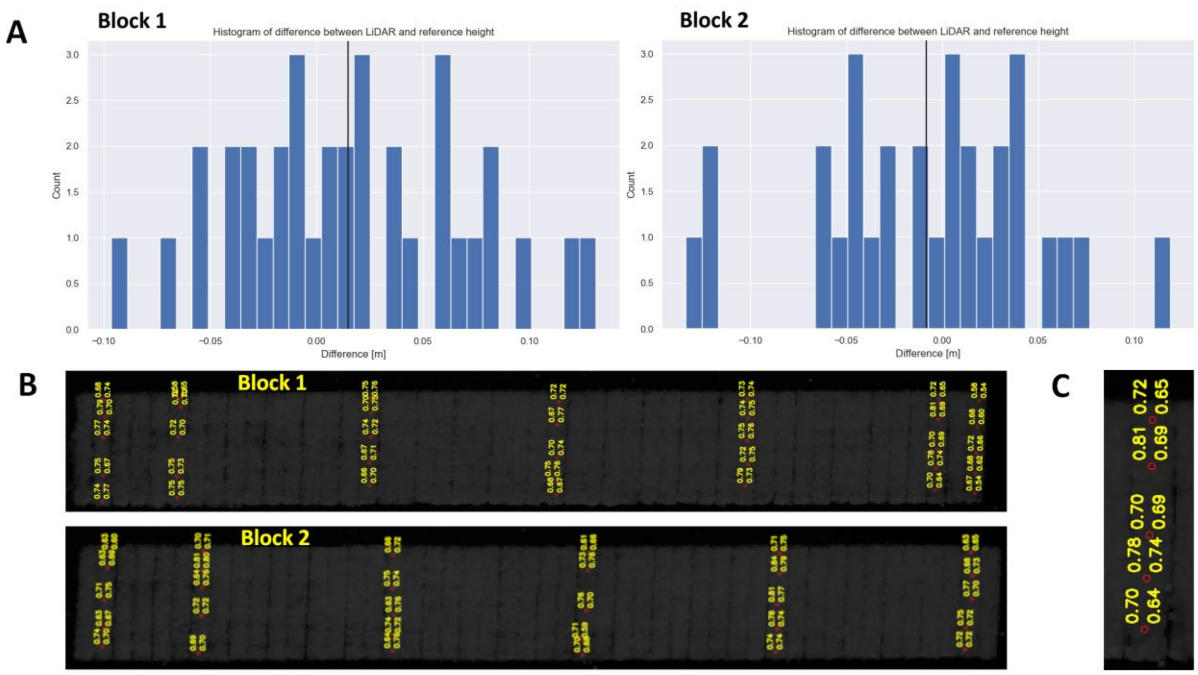

Figure 5 Manual validation of the ALFA software crop height assessment. A) Distribution of the difference between the software-computed crop height and the manual measurement. Blocks 1 and 2 are shown separately. B) Visualization of the field-plots that where included in the validation, identification of blocks 1 and 2. C) A detailed view of a selected field-plot showing the points of validation. The values in the right column show the software-computed crop height and the particular point. The values in the left column represent the manual measurement.

Dynamical analysis of crop height

Measurement of crop height at a single time-point may not be sufficient in many cases. Plant growth is a dynamic process which is highly non-linear (Cao and Moss, 1989). To assess crop growth, we measure the crop height at multiple time points during the vegetative developmental phase. In our case, we performed 7 UAV scanning flights over 3 experimental blocks with a total of 144 barley field-plots. Seven scanning flights were performed between April and June 2021. Scanning was performed in automatic flight mode at 20 m AGL height. After necessary pre-processing steps, the point-cloud files were processed by our software (see Implementation). Multiple time-point measurements then allow to visualize growth in various ways: A simple bar graph of crop height at particular time-points, relative growth rates based on crop height data, or parameters of the sigmoid model fitted to the growth curve. Plotting crop height (Fig 6A) is the most straight-forward way but it does not allow for easy comparison of growth dynamics in individual field-plots. This is better visualized by a sigmoid fit of the growth curve (Fig 6C). It shows the overall dynamics of vegetative growth for each plot, but

information about plant performance from individual time-points is hidden. On the contrary, RGR (Fig. 6B) shows the growth rate in particular time-points and helps to understand the overall dynamics of plant growth. The three parameters of the sigmoid (A, B, and C, see section Implementation above) can also be visualized separately by means of a heat map (see Fig. 4).

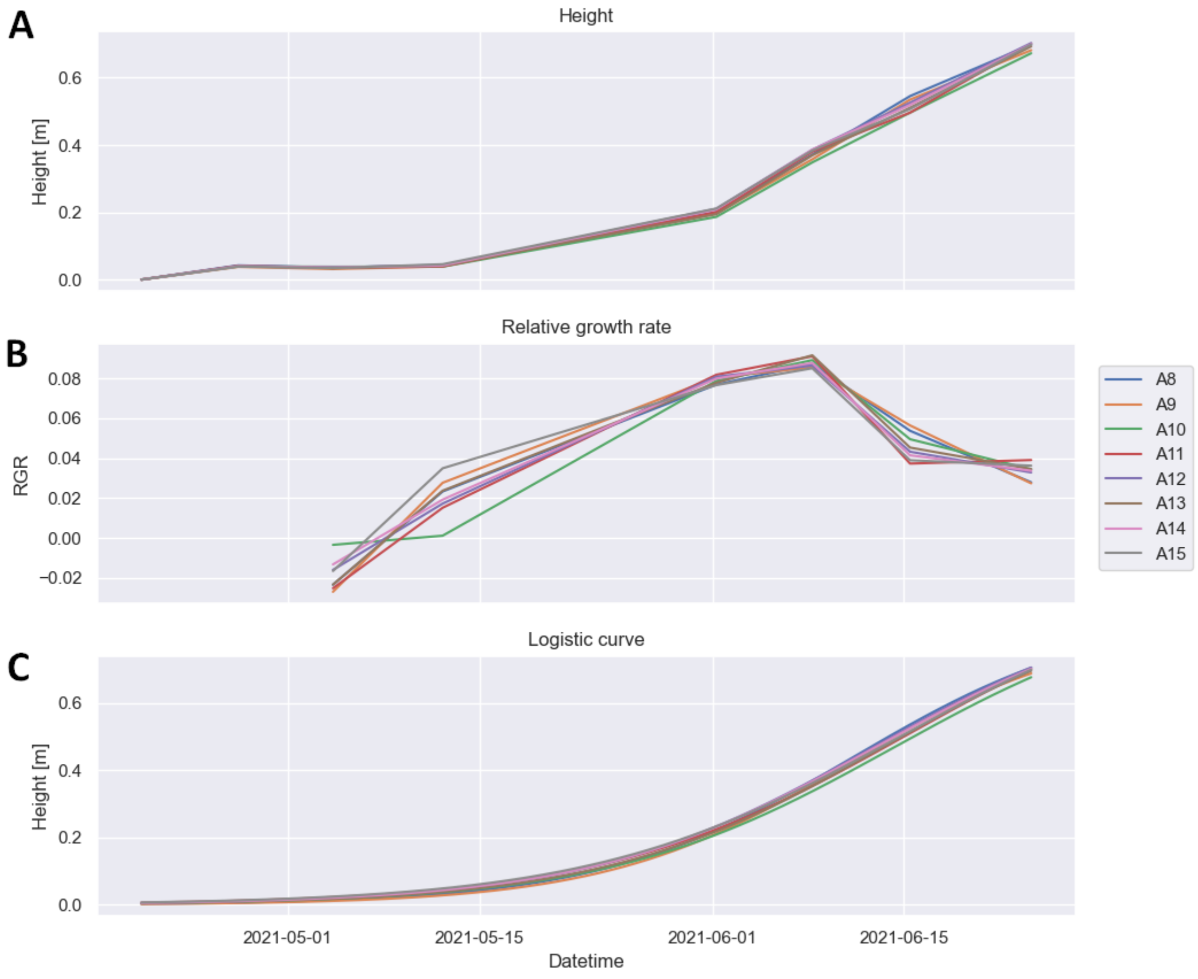

Figure 6 Barley growth during vegetation season in selected representative field-plots. For the individual field-plots, the figure shows the evolution of the crop height (A), RGR (B) and logistic (sigmoid) curves (C).

To test our new software, we evaluated crop growth in 3 blocks, in the total of 144 field-plots. All of the plots contained the same genotype (cv. Francin), sown in a place where we previously observed very heterogeneous growth characteristics in cereals. For this reason, we decided to visualize the growth heterogeneity using a precise measurement of plant growth dynamics. Important differences in growth dynamics are obvious from the RGR and growth curve parameters (Fig 6B, C). The heterogeneity is particularly visible in the heat map (Fig 4) of the sigmoid growth model parameters.

The heat map shows "patterns" of spatially correlated growth differences. It is known that barley is sensitive to different soil compaction that significantly affects root and shoot growth (Mulholland et al., 1996). Since chemical analysis did not reveal any important variability in chemical and nutritional composition of soil (data not shown), we hypothesize that the physical properties of the soil play the key role.

CONCLUSION

The aim of the software development was to design and optimize an algorithm capable of fast extraction of crop height from point-clouds obtained by UAV LiDAR scanning. The ALFA software (available freely for non-commercial use here: https://github.com/frycaktadeas/alfa) uses point-cloud data, converts them into a digital image, and extracts the trait of interest. The entire analysis of 144 field plots (1.2 x 9 meters each) measured at 7 time points takes about 3 minutes at a standard PC. The RMSD difference between the ALFA software computed crop height (from UAV LIDAR data) and a manual measurement (by a ruler) was 5.7 cm. Three different ways of expressing the plant growth dynamics are introduced to allow for understanding the plant growth dynamics and plant reactions to environmental changes. For the future, we plan to add a statistical toolbox to the ALFA software. The toolbox will assess the plant growth dynamic by means of hierarchical Bayesian model that correctly accounts for the spatially correlation of the growth dynamics. In this way, the true effects of treatment can be extracted from the inherently correlated growth data.

Availability of data and materials

Software tool is freely accessible here: https://github.com/frycaktadeas/alfa

The datasets analyzed during the current study are available from the corresponding author on reasonable request.

Competing interests

The authors have declared that no competing interests exist.

Funding

The work was supported by the ERDF project "Plants as a tool for sustainable global development" (No. CZ.02.1.01/0.0/0.0/16_019/0000827).

REFERENCES

Brede, B., Lau, A., Bartholomeus, H.M., Kooistra, L., 2017. Comparing RIEGL RiCOPTER UAV LiDAR derived canopy height and DBH with terrestrial LiDAR. Sensors (Switzerland) 17. https://doi.org/10.3390/s17102371

Cao, W., Moss, D.N., 1989. Temperature Effect on Leaf Emergence and Phyllochron in Wheat and Barley. Crop Sci. 29, 1018–1021. https://doi.org/10.2135/CROPSCI1989.0011183X002900040038X

French, A.N., Gore, M.A., Thompson, A., 2016. Cotton phenotyping with lidar from a track-mounted platform, in: Autonomous Air and Ground Sensing Systems for Agricultural Optimization and Phenotyping. Conference on Autonomous Air and Ground Sensing Systems for Agricultural Optimization and Phenotyping, p. 98660B. https://doi.org/10.1117/12.2224423

GAO, Min, et al. Individual maize location and height estimation in field from UAV-borne LiDAR and RGB images. Remote Sensing, 2022, 14.10: 2292.

GRANSHAW, Stuart I. Photogrammetric terminology. The Photogrammetric Record, 2016, 31.154: 210-252.

Hoffmann, W.A., Poorter, H., n.d. Avoiding Bias in Calculations of Relative Growth Rate. https://doi.org/10.1093/aob/mcf140

Holman, F.H., Riche, A.B., Michalski, A., Castle, M., Wooster, M.J., Hawkesford, M.J., 2016. High throughput field phenotyping of wheat plant height and growth rate in field plot trials using UAV based remote sensing. Remote Sens. 8. https://doi.org/10.3390/rs8121031

HÜTT, Christoph, et al. UAV LiDAR Metrics for Monitoring Crop Height, Biomass and Nitrogen Uptake: A Case Study on a Winter Wheat Field Trial. PFG–Journal of Photogrammetry, Remote Sensing and Geoinformation Science, 2022, 1-12.

Iglhaut, J., Cabo, C., Puliti, S., Piermattei, L., O'connor, J., Rosette, J., Uk, A.A., Gutiérrez Quirós, G., 2019. Structure from Motion Photogrammetry in Forestry: a Review. https://doi.org/10.1007/s40725-019-00094-3

JIN, Shichao, et al. Deep learning: individual maize segmentation from terrestrial lidar data using faster R-CNN and regional growth algorithms. Frontiers in plant science, 2018, 9: 866.

KOCH, Barbara; HEYDER, Ursula; WEINACKER, Holger. Detection of individual tree crowns in airborne lidar data. Photogrammetric Engineering & Remote Sensing, 2006, 72.4: 357-363.

LI, Wenkai, et al. A new method for segmenting individual trees from the lidar point cloud. Photogrammetric Engineering and Remote Sensing, 2012, 78.1: 75-84.

LIU, Kuan; DONG, Xiaoya; QIU, Baijing. Analysis of cotton height spatial variability based on UAV-LiDAR. International Journal of Precision Agricultural Aviation, 2020, 3.3.

Malambo, L., Popescu, S.C., Murray, S.C., Putman, E., Pugh, N.A., Horne, D.W., Richardson, G., Sheridan, R., Rooney, W.L., Avant, R., Vidrine, M., McCutchen, B., Baltensperger, D., Bishop, M., 2018. Multitemporal field-based plant height estimation using 3D point clouds generated from small unmanned aerial systems high-resolution imagery. Int. J. Appl. Earth Obs. Geoinf. 64, 31–42. https://doi.org/10.1016/j.jag.2017.08.014

MONTZKA, Carsten, et al. Sensitivity of LiDAR Parameters to Aboveground Biomass in Winter Spelt. Drones, 2023, 7.2: 121.

Mulholland, B.J., Black, C.R., Taylor, I.B., Roberts, J. a, Lenton, J.R., 1996. Effect of soil compaction on barley (Hordeum vulgare l) growth .1. possible role for ABA as a root-sourced chemical signal. J. Exp. Bot. 47, 539–549.

OpenCV, L., 2008. Computer vision with the OpenCV library. Gary Bradski, Adrian Kaehler 1–15.

Polák, M., Mirijovský, J., Hernándiz, A.E., Špíšek, Z., Koprna, R., Humplík, J.F., 2021. Innovative uav lidar generated point-cloud processing algorithm in python for unsupervised detection and analysis of agricultural field-plots. Remote Sens. 13. https://doi.org/10.3390/rs13163169

TEN HARKEL, Jelle; BARTHOLOMEUS, Harm; KOOISTRA, Lammert. Biomass and crop height estimation of different crops using UAV-based LiDAR. Remote Sensing, 2019, 12.1: 17.

TJØRVE, Even. Shapes and functions of species–area curves: a review of possible models. Journal of Biogeography, 2003, 30.6: 827-835.

WANG, Han, et al. Maize Ear Height and Ear–Plant Height Ratio Estimation with LiDAR Data and Vertical Leaf Area Profile. Remote Sensing, 2023, 15.4: 964.

WANG, Yunsheng, et al. Lidar point cloud based fully automatic 3D single tree modelling in forest and evaluations of the procedure. Int. Arch. Photogramm. Remote Sens. Spat. Inf. Sci, 2008, 37.Part B6b: 45-51.

Zhu, Y., Sun, G., Ding, G., Zhou, Jie, Wen, M., Jin, S., Zhao, Q., Colmer, J., Ding, Y., Ober, E.S., Zhou, Ji, 2021. Large-scale field phenotyping using backpack LiDAR and CropQuant-3D to measure structural variation in wheat. Plant Physiol. 187, 716–738. https://doi.org/10.1093/plphys/kiab324
