# Supplementary material for: Crop growth dynamics: Fast automatic analysis of LiDAR images in field-plot experiments by specialized software ALFA
Source: PLoS One. 2024 Jan 18;19(1):e0297153. doi: 10.1371/journal.pone.0297153 (PMC10796001; doi:10.1371/journal.pone.0297153)
Supplement: Attachment — Submitted filename: PONE-D-23-28678.docx [file pone.0297153.s002.docx]

**Manuscript Number: PONE-D-23-28678**

Title: ALFA - Airborne LiDAR Field-plot Analysis. Fast automatic point-cloud filtering algorithm for analysis of 3 crop growth dynamics in field-plot experiments

**Dear editor**

The manuscript is potentially a publishable study, and is related to the journal.

But the discussion is not well prepared. The scientific issues of the paper are not focused, mostly data representation, and the discussion part is not well presented.

The main objectives of the current study are to extract the information on crop height at many individual cereal field-plots at multiple time points

This subject is crucial for smart agriculture. However, there are some remarks that should be taken into consideration. The discussion must to be supported by earlier researchers and consider any peer review of the findings of previous studies. So, it could hardly attract the international readers interests.

**The abstract**

The authors should focus on improving your abstract by adding some your key results such as

**In the line 24**

You can add this sentence: The entire analysis of 144 field plots measured at 7 time points takes about 3 minutes at a standard PC. it should be included.

Also, you should add the key finding of validation part.

Please include the research problem in abstract section.

**The introduction**

- I appreciate the introduction. It was well-written. However, in the 3 first sentence needs references.
- The introduction is beautifully written. I appreciate it. However, the most recent studies should be included in the literature review section. Please review the most recent publications, such as those published between 2017 and 2022, to identify any research gaps.

**2.The materials and methods**

- Please, enhance the section of materials and methods**.** I recommended confirm the references of all methods.
- Please, you should numbering the equations.

**3. Results and Discussion**

- Please the description of your results and the presentation of your results should be improved.
- Expanding your discussion will help your paper flow better. By analyzing your finding, connecting it to recent research, and describing the main results in your discussion, you can further expand your argument
- Answering why is this manuscript important?
- Greatly improve the mechanistic arguments about dynamical analysis. In general, some mechanistic arguments are not well developed.

**4. CONCLUSION**

- I advise to provide comprehensive evaluation and recommendations for further study, which are required in the paper's conclusion. if the discussion and the findings are kept separate. It will get better.
- Rethink to improve the quality of each figure and the design of the table.

**Other comments:**

- Please include your study's hypothesis along with the data statistical analysis.
- The statistical analysis can’t satisfy your study because there is no statistical analysis.
- The study seems only optimizing an algorithm of fast extraction of crop height. It could hardly attract the international readers interests. I recommend avoiding let your manuscript like a case report.
