# Supplementary material for: Crop growth dynamics: Fast automatic analysis of LiDAR images in field-plot experiments by specialized software ALFA
Source: PLoS One. 2024 Jan 18;19(1):e0297153. doi: 10.1371/journal.pone.0297153 (PMC10796001; doi:10.1371/journal.pone.0297153)
Supplement: Attachment — Submitted filename: Response to Reviewers_PONE-D-23-28678.docx [file pone.0297153.s003.docx]

**Response to Reviewers**

Dear editor and reviewers,

thank you for your valuable comments, which we believe have significantly improved the quality of our manuscript. Our methodological/software article is mainly aimed at the broad community of agricultural researchers and crop breeders. We believe that our software will make it more accurate, faster and easier for them to evaluate their small-plot field trials. In our paper, we detail both the architecture and the testing and optimization capabilities of the software to automatically evaluate the height parameter for each of the scanned plots. Thus, the present paper can serve not only as an instruction manual but also as an inspiration for further development of similar software for developers of laser scanning software in agriculture, which is currently scarce and very expensive. The text of the manuscript has been substantially revised and details of the changes made can be found in your comments below and also in the tracked changes version of the manuscript. We hope that the changes made will meet your expectations and allow the manuscript to be published.

Sincerely

Jan F. Humplík, corresponding author

**Reviewer and editor comments**

**Editor**

1. The title should be rewritten.

*Thank you for your valuable comments. The title has been changed according recommendation.*
2. Improve the abstract by adding some your key results.

*Thank you. We add key results and software features to the abstract.*
3. The most recent studies should be included in the literature review section.

*The Introduction section has been enriched for most recent references. However, we have tried to keep the Introduction section as straightforward and understandable as possible. Therefore, we refer those interested in a detailed analysis directly to the high-quality comprehensive review by Rivera et al. 2023.*

4. The description and the presentation of your results should be improved.

*The results and discussion section has been split into self-contained chapters and substantially rewritten. Also, Figures 1 and 2 have been revised as recommended by the reviewers.*

5. I can not see the difference before and after the affine correction, clarify it.

*The description of Figure 3 has been changed to clearly explain the process of distortion correction. Whereas in raw image the plots do not have a vertical boundaries, affine correction fix this issue to make rectangular shapes.*

7. Include your study's hypothesis.

*This paper is methodological/software paper, for this reason we do not propose any hypothesis or biological question. On the other hand, publishing this software and make it freely available, may help to test the hypotheses of many researchers worldwide.*

8. Avoid let your manuscript like a case report.

*Our study aims mainly to develop and test software for LiDAR data processing that is lacking in biology/agricultural research community as obvious from systematic review of Rivera et al., 2023. For this reason our paper do not describe case study, but design of novel software solution. This may help to the community not only to use the software, but also develop new generation of similar software or adopt it as core of new software solution.*

**Reviewer #1:**

The paper has been meticulously composed, proficiently elucidating the robustness of the employed algorithm, the requisite dataset, and the strategies for effective plant height optimization. The authors could highlight the limitations and complexities associated with the implementation of the UAV LiDAR system. Additionally, they should outline their intended measures for enhancing user-friendliness, particularly for farmers with fundamental computer skills.

*Thank you for your valuable comments. We add commentary to the UAV-LiDAR implementation into Introduction section according your comment. Also we note in the Conclusion section that we plan to develop statistical toolbox that may help to the users analyze their data directly in the software. However, there will be still certain level of computer skill needed to use the software. On the other hand the software is mainly intended for Agricultural Research and Breeding stations, where this level of computer skills may be expected.*

**Reviewer #2:**

1. The title should be rewritten.

*Thank you for your valuable comments. The title has been changed according recommendation.*
2. Selection of Region of Interest at line 101 should be corrected.

*Thank you. This part has been rewritten according your recommendation.*

3. Figure 2 is not clear.

*Thank you, Figure 2 and its description were revised to provide clear message. Hopefully, it satisfied your comment.*

4. I can not see the difference before and after the affine correction, clarify it.

*The description of Figure 3 has been changed to clearly explain the process of distortion correction. Whereas in raw image the plots do not have a vertical boundaries, affine correction fix this issue to make rectangular shapes.*

5. In line 206, heterogeneity is particularly visible in the heat map (Fig 4) of the sigmoid growth model parameters. Discuss and clarify the heat map.

*Thank you. This issue is discussed from the line 279 in Discussion part. We revised this part to clarify the heterogeneity in the field. As stated in Discussion it may be caused by different physical properties in the soil, for which spring barley is very sensitive.*

**Reviewer #3:**

**Manuscript Number: PONE-D-23-28678**

Title: ALFA - Airborne LiDAR Field-plot Analysis. Fast automatic point-cloud filtering algorithm for analysis of 3 crop growth dynamics in field-plot experiments

**Dear editor**

The manuscript is potentially a publishable study, and is related to the journal.

But the discussion is not well prepared. The scientific issues of the paper are not focused, mostly data representation, and the discussion part is not well presented.

*Thank you for your valuable comments. The results and discussion section has been split into self-contained chapters and substantially rewritten. Also, Figures 1 and 2 have been revised as recommended by the reviewers. Discussion part was rewritten, new references were added to cover complexity of data analysis mainly from point of view of dynamics of plant growth.*

The main objectives of the current study are to extract the information on crop height at many individual cereal field-plots at multiple time points

This subject is crucial for smart agriculture. However, there are some remarks that should be taken into consideration. The discussion must to be supported by earlier researchers and consider any peer review of the findings of previous studies. So, it could hardly attract the international readers interests.

*Thank you. Our software is mainly intended for agricultural research where the field-plot system is common. Specialized software for LiDAR data processing that is lacking in biology/agricultural research community as obvious from systematic review of Rivera et al., 2023. Publishing it in open journal may help to the community not only to use the software, but also develop new generation of similar software or adopt it as core of new software solution for different purposes than described in the paper. For these reasons we believe that it may attract readers interest more easily than some really specialized enclosed study.*

**The abstract**

The authors should focus on improving your abstract by adding some your key results such as

*Thank you. We add key results and software features to the abstract.*

**In the line 24**

You can add this sentence: The entire analysis of 144 field plots measured at 7 time points takes about 3 minutes at a standard PC. it should be included.

*Thank you. It has been included according your recommendation.*

Also, you should add the key finding of validation part.

*Key findings were noted in the Abstract.*

Please include the research problem in abstract section.

*Thank you. Main research problem was lacking useful software solution that is noted in the Abstract now.*

**The introduction**

- I appreciate the introduction. It was well-written. However, in the 3 first sentence needs references.

*Thank you for your comment. We add references according your recommendation.*

- The introduction is beautifully written. I appreciate it. However, the most recent studies should be included in the literature review section. Please review the most recent publications, such as those published between 2017 and 2022, to identify any research gaps.

*The Introduction section has been enriched for most recent references. However, we have tried to keep the Introduction section as straightforward and understandable as possible. Therefore, we refer those interested in a detailed analysis directly to the high-quality comprehensive recent review by Rivera et al. 2023.*

**2.The materials and methods**

- Please, enhance the section of materials and methods**.** I recommended confirm the references of all methods.

*The original Implementation section has been rearranged, rewritten and renamed Materials and Methods. New literature references have been added.*

- Please, you should numbering the equations.

*Equations were numbered according your recommendation.*

**3. Results and Discussion**

- Please the description of your results and the presentation of your results should be improved.

*The results and discussion section has been split into self-contained chapters and substantially rewritten. Also, Figures 1 and 2 have been revised as recommended by the reviewers.*

- Expanding your discussion will help your paper flow better. By analyzing your finding, connecting it to recent research, and describing the main results in your discussion, you can further expand your argument

*Discussion section has been substantially rewritten, new references were added and most important topics are newly discussed.*

- Answering why is this manuscript important?

*Specialized software for LiDAR data processing is lacking in biology/agricultural research community as obvious from systematic review of Rivera et al., 2023. Publishing it in open journal may help to the community not only to use the software, but also develop new generation of similar software or adopt it as core of new software solution for different purposes than described in the paper. For these reasons we believe that publishing manuscript with our software may importantly affect the research in this field.*

- Greatly improve the mechanistic arguments about dynamical analysis. In general, some mechanistic arguments are not well developed.

*The Discussion part has been importantly rewritten and topic about modeling of plant growth dynamics was added. We believe that this help to the readers to understand better this important problematics.*

**4. CONCLUSION**

- I advise to provide comprehensive evaluation and recommendations for further study, which are required in the paper's conclusion. if the discussion and the findings are kept separate. It will get better.

*Thank you for your comment. As we noted in the conclusion, the most important future activity connected to the ALFA software will be development of statistical toolbox that will help to users to analyze data directly in the software. For this we need to provide software to the wide community and based on their feedback and experiences toolbox will developed and optimized. The results and discussion section has been split into self-contained chapters and substantially rewritten.*

- Rethink to improve the quality of each figure and the design of the table.

*Thank you. The figures 1 and 2 were modified and quality of figures was improved. In this paper we do not present any table. Hopefully it will satisfy your comments.*

**Other comments:**

- Please include your study's hypothesis along with the data statistical analysis.

*Thank you for your comments. This paper is methodological/software paper, for this reason we do not propose any hypothesis or biological question. On the other hand, publishing this software and make it freely available, may help to test the hypotheses of many researchers worldwide.*

- The statistical analysis can’t satisfy your study because there is no statistical analysis.

*Thank you for your comment. We do not provide any statistical analysis, because we do not test any hypothesis in this methodological/software article. However, anybody who need to accurate compare the growth of cereal or similar field-plots will benefit from the manuscript, because she/he can test their hypothesis based on relevant and accurate data from LiDAR scanning. The statistical analysis of logistic curves is really interesting problem that do not posses straightforward solution as described e.g. in Tsoularis and Wallace (2002). As we noted in the conclusion, the most important future activity connected to the ALFA software will be development of statistical toolbox that will help to users to analyze data directly in the software. This toolbox will be based on Bayesian statistics as we did previously for seedling germination (Kaplan-Meier) curves (Humplík et al., 2020; http://www.bayes4plants.com/).*

- The study seems only optimizing an algorithm of fast extraction of crop height. It could hardly attract the international readers interests. I recommend avoiding let your manuscript like a case report.

*Thank you. Our software is mainly intended for agricultural research where the field-plot system is common. Specialized software for LiDAR data processing that is lacking in biology/agricultural research community as obvious from systematic review of Rivera et al., 2023. Publishing it in open journal may help to the community not only to use the software, but also develop new generation of similar software or adopt it as core of new software solution for different purposes than described in the paper. For these reasons we believe that it may attract readers interest more easily than some really specialized enclosed study.*

Tsoularis, A., & Wallace, J. (2002). Analysis of logistic growth models. *Mathematical biosciences*, *179*(1), 21-55.
